# Sarilumab plus methotrexate suppresses circulating biomarkers of bone resorption and synovial damage in patients with rheumatoid arthritis and inadequate response to methotrexate: a biomarker study of MOBILITY

**DOI:** 10.1186/s13075-016-1132-9

**Published:** 2016-10-06

**Authors:** Anita Boyapati, Jérôme Msihid, Stefano Fiore, Janet van Adelsberg, Neil M. H. Graham, Jennifer D. Hamilton

**Affiliations:** 1 Regeneron Pharmaceuticals, Inc., 777 Old Saw Mill River Road, Tarrytown, NY 10591 USA; 2 Sanofi R&D, 1 Avenue Pierre Brossolette, 91380 Chilly-Mazarin, France; 3 Sanofi US, 55 Corporate Drive, Bridgewater, NJ 08807 USA

**Keywords:** Sarilumab, Rheumatoid arthritis, Biomarker, RANKL/OPG

## Abstract

**Background:**

Interleukin 6 (IL-6) signaling plays a key role in the pathophysiology of rheumatoid arthritis (RA) and is inhibited by sarilumab, a human monoclonal antibody blocking the IL-6 receptor alpha (IL-6Rα). The effects of sarilumab plus methotrexate (MTX) on serum biomarkers of joint damage and bone resorption were assessed in two independent studies (phase II (part A) and phase III (part B)) of patients with RA with a history of inadequate response to MTX from the MOBILITY study (NCT01061736).

**Methods:**

Serum samples were analyzed at baseline and prespecified posttreatment time points. Biomarkers of tissue destruction, cartilage degradation, and synovial inflammation were measured in part A; assessment of these markers was repeated in part B and included additional analysis of biomarkers of bone formation and resorption (including soluble receptor activator of nuclear factor-kB ligand (sRANKL)). A mixed model for repeated measures was used to compare treatment effects on change in biomarkers. Additionally, changes from baseline in biomarkers were compared between American College of Rheumatology 50 % responders and nonresponders and between patients who achieved or did not achieve low disease activity (LDA), separately by treatment group, at week 24.

**Results:**

In part A, sarilumab 150 and 200 mg every 2 weeks (q2w) significantly reduced biomarkers of tissue destruction, cartilage degradation, and synovial inflammation at both 2 and 12 weeks posttreatment (*p* < 0.05 vs placebo). These results were replicated in part B, with markers of these damaging processes reduced at weeks 2 and 24 (*p* < 0.05 vs placebo). Additionally, sarilumab 200 mg q2w significantly reduced both sRANKL and sRANKL/osteoprotegerin ratio at week 24 (*p* < 0.01 vs placebo). Trends for reduction were noted for several biomarkers in patients who achieved LDA compared with those who did not.

**Conclusions:**

Sarilumab plus MTX significantly suppressed biomarkers of bone resorption and joint damage, as compared with placebo plus MTX, in patients with RA. Additional work is needed to determine whether differences in biomarker profiles at baseline or posttreatment can identify patients who achieve improvement in disease activity.

**Trial registration:**

ClinicalTrials.gov, NCT01061736, February 2, 2010.

## Background

Rheumatoid arthritis (RA) is an autoimmune disease characterized by chronic overactivation of the inflammatory system and progressive joint destruction [1]. The localized joint symptoms observed in RA result from persistent synovial inflammation associated with damage to articular cartilage and underlying bone [1, 2], which may lead to progressively impaired function and disability [3].

Both innate and adaptive immune processes mediated by cytokine activity play a role in the pathophysiology of RA [4]. For example, the concentration of interleukin 6 (IL-6) is increased in the serum and synovial fluid of patients with RA relative to healthy individuals [5] and correlates with disease activity and joint destruction [4]. Elevation of IL-6 concentrations in joints may facilitate synovial fibroblast activation, and bone resorption and joint damage, through osteoclast formation [6, 7]. The combination of reduced bone formation and increased bone resorption is a characteristic feature of RA [8].

Studies in cell cultures and mouse models have demonstrated the critical role of IL-6 in the induction of bone-resorptive factors (e.g., receptor activator of nuclear factor-kB ligand (RANKL)) and joint-destructive proteins (e.g., matrix metalloproteinases (MMPs)) from osteoclasts and fibroblast-like synoviocytes (FLS) [6, 9–12]. RANKL, which exists in membrane-bound and soluble forms (sRANKL), binds to RANK to induce osteoclast formation, survival, fusion, and activation [13]. Blockade of the IL-6 receptor (IL-6R) inhibits osteoclast formation in vitro and in vivo [14], and the induction of RANKL observed in a collagen-induced arthritis monkey model is suppressed by treatment with the IL-6R antibody tocilizumab [15].

IL-6R inhibition also blunts RANKL production in FLS from patients with RA [9]. Osteoprotegerin (OPG), a decoy receptor for RANKL, binds both forms of RANKL, preventing activation of RANK and inhibiting osteoclastogenesis [13]. The RANKL/OPG ratio regulates the balance between bone turnover and bone formation, with a higher ratio favoring enhanced bone resorption [13, 16]. The formation of type I collagen fragments, such as carboxy-terminal collagen crosslinks 1 (CTX-1), another indicator of bone turnover, is elevated in patients with RA with joint destruction and radiographic progression compared with controls [8, 17]. IL-6 signaling may also influence levels of serum osteocalcin (OC), a marker of bone formation, further suggesting that modulation of this pathway may positively impact the balance of bone turnover and formation [18].

Articular inflammation also leads to the secretion of joint-destructive enzymes, (e.g., MMPs) by rheumatoid synovial fibroblasts [19]; thus, MMP substrates can be used as biomarkers of articular damage [18]. MMP-cleaved fragments derived from collagens or the acute-phase reactant C-reactive protein (CRP) have been described in patients with established RA [18]. Collagen types I, II, and III are the major components of bone, cartilage, and synovium, respectively [20], and MMP-cleaved fragments (C1M, C2M, and C3M, respectively) may reflect articular remodeling [20, 21]. An MMP-cleaved fragment of CRP, CRPM, is also a measure of synovial inflammation [18, 22, 23].

Sarilumab is a human monoclonal antibody directed against both membrane-bound and soluble forms of IL-6Rα [24]. Sarilumab blockade of IL-6 binding to IL-6Rα results in inhibition of IL-6–mediated signal transduction [25]. The efficacy and safety of sarilumab in combination with methotrexate (MTX) in patients with moderate-to-severe RA and inadequate response to MTX (MTX-IR) were evaluated in the two-part (phase II (part A) and phase III (part B)) MOBILITY trial (NCT01061736) [24, 26]. In MOBILITY A and B, patients treated with sarilumab demonstrated statistically significant improvements in American College of Rheumatology 20 % (ACR20) response rate at weeks 12 and 24, respectively. In MOBILITY B, patients treated with sarilumab also demonstrated significant improvements in least squares mean change in the Health Assessment Questionnaire–Disability Index (HAQ-DI) at week 16 and mean change in the van der Heijde modified total Sharp score (mTSS) at week 52, relative to placebo + MTX. The erosion score (ES) and joint space narrowing (JSN), components of the mTSS, were significantly reduced compared with placebo + MTX as early as week 24. Sarilumab also reduced serum levels of CRP, a marker of inflammation commonly assessed in patients with RA. Both doses of sarilumab were generally well-tolerated, and the most common treatment-emergent adverse events included infections, neutropenia, injection site reactions, and increased transaminases.

To better understand the mechanism through which sarilumab inhibits progression of structural damage (JSN and ES), we assessed biomarkers indicative of joint damage and bone resorption. First, biomarkers of bone and tissue destruction and synovial inflammation were measured in patients from the dose-ranging MOBILITY part A study. These analyses were then replicated and expanded upon (by including biomarkers of bone formation and resorption) in patients from the MOBILITY part B study.

## Methods

### Study design

The results of MOBILITY (NCT01061736), a two-part (phase II (part A) and phase III (part B)), randomized, double-blind, placebo-controlled, multicenter study that evaluated the efficacy and safety of subcutaneous sarilumab in combination with MTX in patients with active RA and MTX-IR have previously been described [24, 26]. Part A of MOBILITY was a 12-week, phase II, dose-ranging study in patients with active RA who were randomized to receive MTX in combination with placebo or one of five subcutaneous sarilumab doses [26]. Patients who participated in part A were not eligible for part B, a 52-week, phase III study evaluating the safety and efficacy of sarilumab 150 mg and 200 mg every 2 weeks (q2w) in combination with MTX [24].

The protocol was approved by the appropriate ethics committees/institutional review boards (see “Acknowledgments” for details), and all patients provided written informed consent before study entry. The study was conducted in compliance with institutional review board regulations, the International Conference on Harmonisation Guidelines for Good Clinical Practice, and the Declaration of Helsinki.

### Sera collection

In part A, biomarkers were measured retrospectively in sera collected at baseline (i.e., before receiving the first treatment dose), and at 2 and 12 weeks posttreatment, from patients receiving placebo + MTX (n = 45), sarilumab 150 mg q2w + MTX (n = 46), or sarilumab 200 mg q2w + MTX (n = 45). These doses were chosen for the present analyses as they were selected for additional efficacy and safety analyses in MOBILITY part B. Sera were collected under fasting conditions at baseline and week 12 and under nonfasting conditions at week 2. Patients were included in the analyses if at least one baseline value and at least one postbaseline value were available for one or more biomarkers under evaluation.

Biomarker analyses from part A were replicated and expanded upon in part B in sera collected under fasting conditions at baseline and 2, 24, and 52 weeks posttreatment from randomly selected patients receiving placebo + MTX (n = 128) or sarilumab 200 mg q2w + MTX (n = 131). Sarilumab 200 mg q2w was chosen for these analyses because this dose demonstrated better efficacy compared with sarilumab 150 mg q2w with respect to the bone and joint x-ray outcomes (i.e., mTSS, ES, and JSN) in MOBILITY part B, and the additional biomarkers measured reflect pathological processes associated with these scores. To be selected for this retrospective biomarker analysis, patients were required to have baseline, week 2, and week 24 biomarker samples and week 24 radiographic data available. Patients were included in the analyses if the baseline value and at least one postbaseline value were available for at least 1one biomarker under evaluation.

Starting at week 16 in MOBILITY part B, patients with a lack of efficacy could be “rescued” by switching to open-label sarilumab 200 mg q2w + MTX. Patients who were rescued continued in the study according to their planned visit schedule. Samples drawn from patients in the placebo + MTX group before rescue medication were included in the biomarker analysis. Serum samples obtained from patients in the placebo + MTX group after rescue medication were excluded from the analysis.

### Biochemical marker assays

Retrospective analysis of serum concentrations of C1M, C2M, C3M, and CRPM from patients from MOBILITY part A were measured at Synarc (BioClinica Laboratory, Lyon, France) using a validated proprietary enzyme-linked immunosorbent assay (ELISA) by Nordic Bioscience (Herlev, Denmark). The intra-assay and inter-assay variation (coefficients of variation (CVs)) were <13.8 % for C1M, <19.8 % for C2M, <16.4 % for C3M, and <14.2 % for CRPM. Serum concentrations of C1M, C2M, C3M, MMP-3, CTX-1, and OC from patients from MOBILITY part B were measured at Nordic Bioscience using a validated ELISA (Nordic Bioscience; all CVs <15 %). Serum MMP-3 (Quantikine total MMP-3 (R&D Systems, Minneapolis, MN, USA); CV <10 %) and serum CTX-1 (CV <3.4 %) were measured using the β-CrossLaps (Roche, Basel, Switzerland) assay. Osteocalcin was measured using the validated N-MID-OC kit (Roche; CV <4.6 %). Serum concentrations of sRANKL (human sRANKL ELISA (BioVendor, Brno, Czech Republic)) and OPG (human OPG ELISA (BioVendor)) were measured using validated assays at Pacific Biomarkers (Seattle, WA, USA).

### Statistical analysis

Patient baseline demographics and disease parameters are presented as mean (± standard deviation). Given the non-normal distribution of several biomarkers, median serum concentrations (quartile 1 to quartile 3 interval) were reported for baseline measures.

To evaluate differences in pharmacodynamic changes between sarilumab + MTX and placebo + MTX, a mixed-effect model with repeated measures (MMRM) was performed on rank-transformed percent change from baseline (analysis of variance (ANOVA)-type method), with the treatment, visit, and treatment-by-visit interaction included as fixed effects. Given the similar baseline biomarker values in each treatment group, baseline biomarker values were not included in the model. An MMRM was also performed on the log-transformed sRANKL/OPG ratio (to yield a normal distribution) with treatment, visit, and treatment-by-visit interaction as fixed effects, and baseline biomarker value and baseline biomarker-value-by-visit interaction as fixed covariates. An unstructured covariance structure was assumed in all models. The Bonferroni correction was used to adjust *P* values for multiplicity. A *P* value <0.05 after adjustment was considered significant.

For exploratory purposes, percent changes from baseline in biomarkers and sRANKL/OPG were also compared between responders and nonresponders (patients who achieved or did not achieve ACR50 or low disease activity (LDA), as measured by 28-joint disease activity score by CRP (DAS28-CRP) <3.2) at week 24 using similar methods and after adjustment for baseline values, separately by treatment group; nominal *P* values are reported. Analyses were performed using SAS^®^ v9.2 or higher (SAS Institute, Cary, NC, USA).

## Results

### Patient demographics, disease parameters, and baseline biomarker serum concentrations

Baseline disease characteristics in the biomarker analyses were similar to those in the overall study [24, 26]. In part A (Table 1), the mean age of patients across all treatment groups in these biomarker analyses was 51.0 ± 13.1 years, and patients had a mean RA duration of 7.2 ± 7.3 years. Patients across all treatment groups displayed similar baseline disease characteristics, including tender joint count (27.7 ± 16.2), swollen joint count (17.7 ± 10.8), and CRP concentration (3.0 ± 3.4 mg/dL). In part B (Table 2), the mean age of patients across all treatment groups in these biomarker analyses was 50.2 ± 11.5 years, and patients had a mean RA duration of 8.6 ± 7.5 years. Patients across all treatment groups displayed similar baseline disease characteristics, including tender joint count (26.6 ± 14.7), swollen joint count (16.2 ± 9.4), CRP concentration (1.9 ± 2.0 mg/dL), and mTSS (48.8 ± 66.3). Median baseline serum concentrations of all assayed biomarkers were generally comparable across treatment groups in part A (Table 1) and part B (Table 2).

**Table 1:** Patient demographics, disease parameters, and baseline biomarker serum concentrations from MOBILITY part A biomarker analysis

	Placebo + MTX (n = 45)	Sarilumab 150 mg q2w + MTX (n = 46)	Sarilumab 200 mg q2w + MTX (n = 45)	Total^a^ (n = 136)
Baseline demographic and disease parameters				
Age, mean ± SD, years	54.7 ± 13.1	49.8 ± 12.7	48.4 ± 12.8	51.0 ± 13.1
Sex, female, %	75.6	84.8	80.0	80.1
Duration of RA, mean ± SD, years	8.0 ± 8.6	7.1 ± 6.7	6.4 ± 6.4	7.2 ± 7.3
Anti-CCP antibody positive, %^b^	73.7	95.0	90.0	86.4
Rheumatoid factor positive, %	66.7	87.0	88.9	80.9
Tender joint count, mean ± SD	27.9 ± 17.0	28.1 ± 17.2	26.9 ± 14.6	27.7 ± 16.2
Swollen joint count, mean ± SD	17.6 ± 12.3	18.3 ± 10.9	17.2 ± 9.3	17.7 ± 10.8
CRP, mean ± SD, mg/dL	2.8 ± 2.8	2.6 ± 2.8	3.4 ± 4.4	3.0 ± 3.4
Baseline biomarker serum concentrations, median (quartile 1/quartile 3)				
C1M, ng/mL	198.1 (132.0/263.4)	179.6 (140.2/235.8)	172.3 (132.4/273.0)	179.6 (133.5/259.3)
C2M, ng/mL	0.2 (0.2/0.3)	0.3 (0.2/0.3)	0.2 (0.2/0.4)	0.2 (0.2/0.3)
C3M, ng/mL	45.6 (38.9/58.1)	47.6 (38.3/60.2)	47.9 (37.9/59.3)	47.5 (38.3/59.0)
CRPM, ng/mL	17.3 (12.1/21.7)	16.5 (14.1/21.4)	16.3 (13.9/22.3)	16.7 (13.1/21.7)

^a^All patients receiving placebo, sarilumab 150 mg q2w, or sarilumab 200 mg q2w. ^b^Results not available for the entire biomarker population. *C1M* collagen type I MMP-cleaved fragment, *C2M* collagen type II MMP-cleaved fragment, *C3M* collagen type III MMP-cleaved fragment, *CCP* cyclic citrullinated peptide, *CRPM* C-reactive protein MMP-derived fragment, *MMP* matrix metalloproteinase, *MTX* methotrexate, *q2w* every 2 weeks, *RA* rheumatoid arthritis, *SD* standard deviation

**Table 2:** Patient demographics, disease parameters, and baseline biomarker serum concentrations from MOBILITY part B biomarker analysis

	Placebo + MTX (n = 128)	Sarilumab 200 mg q2w + MTX (n = 131)	Total^a^ (n = 259)
Baseline demographic and disease parameters			
Age, mean ± SD, years	51.1 ± 10.6	49.3 ± 12.3	50.2 ± 11.5
Sex, female, %	77.3	84.7	81.1
Duration of RA, mean ± SD, years	9.1 ± 8.2	8.1 ± 6.7	8.6 ± 7.5
Anti-CCP antibody positive, %	82.8	87.0	84.9
Rheumatoid factor positive, %	87.5	87.8	87.6
Tender joint count, mean ± SD	27.3 ± 14.8	25.9 ± 14.5	26.6 ± 14.7
Swollen joint count, mean ± SD	15.8 ± 8.0	16.6 ± 10.6	16.2 ± 9.4
CRP, mean ± SD, mg/dL	1.7 ± 1.9	2.1 ± 2.1	1.9 ± 2.0
mTSS, mean ± SD	51.8 ± 72.1	45.9 ± 60.2	48.8 ± 66.3
Baseline biomarker serum concentrations, median (quartile 1/quartile 3)			
C1M, ng/mL	114.0 (77.0/175.7)	120.5 (86.1/196.3)	119.6 (80.7/184.2)
C2M, ng/mL	0.3 (0.2/0.4)	0.3 (0.2/0.4)	0.3 (0.2/0.4)
C3M, ng/mL	43.1 (34.6/58.0)	45.4 (34.4/60.5)	44.2 (34.5/59.9)
CTX-1, ng/mL	0.4 (0.3/0.6)	0.4 (0.3/0.5)	0.4 (0.3/0.5)
MMP-3, ng/mL	41.9 (24.6/77.6)	38.9 (21.3/68.7)	40.3 (22.3/73.1)
OC, ng/mL	18.3 (13.0/25.0)	18.6 (14.6/24.7)	18.5 (13.5/24.7)
OPG, pmol/L	4.9 (3.9/6.3)	5.4 (3.9/6.7)	5.2 (3.9/6.5)
sRANKL, pmol/L	1012.5 (385.0/3893.0)	1096.0 (393.0/2161.5)	1026.0 (387.0/2748.5)
sRANKL/OPG	245.1 (64.4/836.5)	186.3 (71.8/401.2)	212.6 (70.8/509.7)

^a^All patients receiving placebo or sarilumab 200 mg q2w. *C1M* collagen type I MMP-cleaved fragment, *C2M* collagen type II MMP-cleaved fragment, *C3M* collagen type III MMP-cleaved fragment, *CCP* cyclic citrullinated peptide, *CRP* C-reactive protein, *CTX-1* carboxy-terminal collagen crosslinks 1, *MMP* matrix metalloproteinase, *mTSS* van der Heijde modified total Sharp score, *MTX* methotrexate, *OC* osteocalcin, *OPG* osteoprotegerin, *q2w* every 2 weeks, *RA* rheumatoid arthritis, *SD* standard deviation, *sRANKL* soluble receptor activator of nuclear factor-kB ligand

### Biomarkers of joint inflammation and damage

**Fig. 1:**
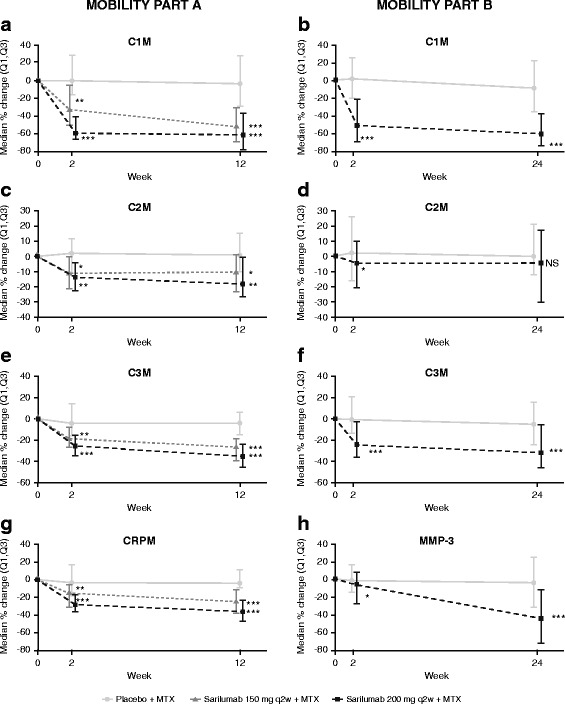
Sarilumab decreases markers of joint damage and inflammation in MOBILITY parts A and B (**a** and **b**, C1M; **c** and **d**, C2M; **e** and **f**, C3M; **g**, CRPM; and **h**, MMP-3). *p < 0.05 vs placebo. **p < 0.01 vs placebo. ***p < 0.0001 vs placebo. *C1M* collagen type I MMP-cleaved fragment, *C2M* collagen type II MMP-cleaved fragment, *C3M* collagen type III MMP-cleaved fragment, *CRPM* C-reactive protein MMP-derived fragment, *MMP-3* matrix metalloproteinase 3, *MTX* methotrexate, *NS* not significant, *(Q1,Q3)* quartile 1 to quartile 3 interval, *q2w* every 2 weeks

Serum concentrations of MMP-generated biomarkers related to joint damage and tissue turnover were measured first in part A (baseline, week 2, and week 12) and subsequently in part B (baseline, week 2, and week 24). In part A, the decrease in serum concentration of these biomarkers from baseline was significantly greater after treatment with sarilumab 150 and 200 mg q2w compared with placebo; suppression was numerically greater with the 200 mg q2w dose compared with the 150 mg q2w dose. The greatest change observed was in C1M, which was significantly suppressed in patients receiving sarilumab relative to patients receiving placebo. Dose-dependent decreases in C1M were observed with sarilumab treatment at week 2 (Fig. 1a); serum concentration of C1M was further suppressed at week 12 in the sarilumab 150 mg q2w group to levels observed in the 200 mg q2w group. A 33.6 % reduction from baseline was observed in the sarilumab 150 mg q2w group at week 2, with a 52.5 % reduction from baseline observed at week 12 (*p* < 0.0001 vs placebo for both time points). In the sarilumab 200 mg q2w group, a 59.4 % reduction from baseline at week 2 and a 61.4 % reduction from baseline at week 12 was observed (*p* < 0.0001 vs placebo at both time points). Treatment with placebo resulted in a 4.1 % decrease from baseline over a 12-week period. In part B, circulating C1M was reduced by 50.1 % at week 2 and 60.3 % at week 24 with sarilumab 200 mg q2w compared with a 2.3 % increase and an 8.1 % reduction from baseline with placebo (*p* < 0.0001 at both time points; Fig. 1b).

Modest changes in the cartilage degradation marker C2M were observed in part A. There was a 0.9 % increase from baseline over the 12 weeks in the placebo group, while sarilumab reduced C2M by >10.0 % by week 2 (sarilumab 150 mg q2w, *p* < 0.05 vs placebo; sarilumab 200 mg q2w, *p* < 0.001 vs placebo; Fig. 1c). This decrease was maintained by sarilumab 150 mg q2w at week 12 (10.2 % decrease from baseline; *p* < 0.05 vs placebo); C2M was further suppressed by sarilumab 200 mg q2w at this time point (18.2 % decrease from baseline; *p* < 0.001 vs placebo). Sarilumab suppression of C2M was less pronounced, and the difference relative to placebo was not observed in part B (Fig. 1d).

In part A, sarilumab 150 mg q2w decreased the synovial inflammation marker C3M by 18.9 % (*p <* 0.001 vs placebo) and 26.6 % (*p* < 0.0001 vs placebo) at weeks 2 and 12, respectively; reductions of 24.6 % (week 2) and 34.9 % (week 12) were observed in the sarilumab 200 mg q2w (*p* < 0.0001 vs placebo at both time points; Fig. 1e). Similar results were observed in part B, in which C3M was reduced by 23.8 % at week 2 and 31.5 % at week 24 (*p* < 0.0001 vs placebo at both time points; Fig. 1f), compared with a 5.3 % reduction over 24 weeks observed with placebo.

Although placebo had minimal effects on CRPM, a marker of synovial inflammation, sarilumab reduced CRPM serum concentrations relative to baseline at weeks 2 and 12 in part A (Fig. 1g). Maximum suppression was observed at week 12 in both sarilumab groups (150 mg q2w, −25.0 % from baseline; 200 mg q2w, −35.8 % from baseline; *p* < 0.0001 vs placebo for both sarilumab groups). In part B, significantly lower serum concentrations of MMP-3, another marker of synovial inflammation, were observed at week 2 with sarilumab 200 mg q2w compared with placebo (−5.4 % from baseline vs −0.4 % from baseline, respectively; *p* < 0.05), and these concentrations were further decreased from baseline by week 24 (−44.2 % vs −2.7 %, respectively; *p* < 0.0001; Fig. 1h).

### Markers of bone resorption

**Fig. 2:**
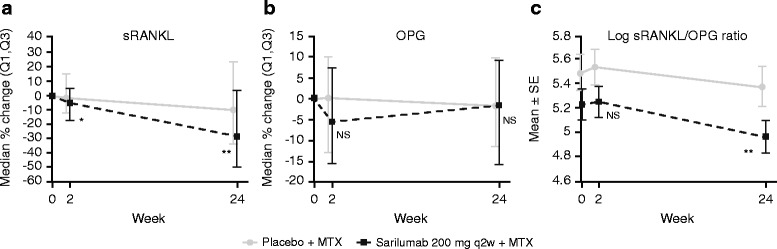
Sarilumab decreases sRANKL, and log RANKL/OPG ratio, markers of bone resorption in MOBILITY part B (**a**, sRANKL; **b**, OPG; **c**, log sRANKL/OPG ratio). *p < 0.05 vs placebo. **p < 0.01 vs placebo. *MTX* methotrexate, *NS* not significant, *OPG* osteoprotegerin, *(Q1,Q3)* quartile 1 to quartile 3 interval, *q2w* every 2 weeks, *RANKL* receptor activator of nuclear factor-kB ligand, *SE* standard error, *sRANKL* soluble RANKL

Serum concentrations of biomarkers related to bone resorption were measured in part B. Sarilumab 200 mg q2w significantly reduced sRANKL relative to placebo at week 2 (*p* < 0.05; Fig. 2a), and sRANKL continued to decrease through week 24 in both groups, with greater suppression observed with sarilumab compared with placebo. At week 24, sarilumab 200 mg q2w significantly suppressed sRANKL more than placebo (−28.6 % vs −10.2 % from baseline, respectively; *p* < 0.01). No significant differences from baseline in OPG were observed in either treatment group at the time points measured (Fig. 2b). However, because of the suppressive effect of sarilumab on sRANKL, a significant decrease in the sRANKL/OPG ratio was observed in the sarilumab 200 mg q2w group compared with placebo (*p* < 0.01) at week 24 (Fig. 2c).

Moderate reductions in CTX-1 were observed at week 24 in the sarilumab 200 mg q2w and placebo groups (−6.7 % and −7.8 % from baseline, respectively) and week 52 (−7.7 % and −7.0 %, respectively), but there were no significant differences between treatment groups at either time point examined (data not shown).

### Marker of bone formation

**Fig. 3:**
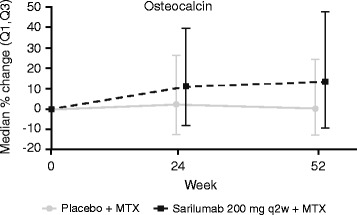
Sarilumab increases OC, a marker of bone formation (nominal *p* = 0.057 vs placebo, week 52). *MTX* methotrexate, *OC* osteocalcin, *(Q1,Q3)* quartile 1 to quartile 3 interval, *q2w* every 2 weeks

Serum concentrations of OC were evaluated at baseline, week 24, and week 52 in samples from part B. Serum OC concentrations remained steady after treatment with placebo over the 52-week study. A numeric trend toward a larger increase in OC was observed with sarilumab 200 mg q2w at week 24 (10.9 %; *p* = 0.107) and at week 52 (13.2 %; *p* = 0.057, unadjusted *p* = 0.029) vs placebo (2.1 % and 0.1 %, respectively), although these results were not significant after adjustment for multiplicity (Fig. 3).

### Biomarker changes by ACR50 response at week 24

Percent change in serum concentrations of biomarkers were examined in ACR50 responders (placebo, n = 34 (26.6 %); sarilumab 200 mg q2w, n = 67 (51.1 %)) and nonresponders (placebo, n = 94 (73.4 %); sarilumab 200 mg q2w, n = 64 (48.9 %)) at week 24 in part B (Table 3). C-reactive protein (a marker of inflammation) and markers of bone resorption and joint damage were assessed. Placebo-treated ACR50 responders demonstrated a greater reduction in CRP from baseline compared with ACR50 nonresponders, although this effect was not observed until week 8 (−30.6 % vs −8.2 %; nominal *p* < 0.05). Only a small difference in the magnitude of CRP suppression was observed between sarilumab responders and nonresponders at this time point (−96.6 % vs −93.3 %; nominal *p* < 0.05).

**Table 3:** Median percent change from baseline in biomarker concentration in ACR50 responder and nonresponder patients at week 24

	Placebo		Sarilumab 200 mg q2w	
	ACR50 responder (n = 34)	ACR50 nonresponder (n = 94)	ACR50 responder (n = 67)	ACR50 nonresponder (n = 64)
CRP				
Week 2	8.5	−3.3	−94.2	−87.5
Week 24	−40.3^**^	−4.2	−96.3	−94.1
C1M				
Week 2	1.6	2.6	−57.1^*^	−43.8
Week 24	−26.7^*^	−7.2	−62.8^**^	−57.0
C2M				
Week 2	0.0	3.4	−4.3	−4.3
Week 24	0.0	3.1	0.0	−6.5
MMP-3				
Week 2	−5.1	1.9	−10.8^*^	−4.4
Week 24	−6.3	−1.4	−50.9	−30.6
OPG				
Week 2	1.7	0.0	−6.0	−2.2
Week 24	0.0	−1.8	−1.1	−2.0
sRANKL				
Week 2	−5.4	−1.2	−7.9	−2.7
Week 24	−23.6^*^	−0.8	−31.4	−24.9

Percent change from baseline in biomarkers transformed in rank was compared between responder and nonresponder patients at week 24 using an analysis of variance (ANOVA)-type method, with response, visit, and response-by-visit interaction as fixed effects, rank-transformed baseline biomarker value and rank-transformed baseline biomarker-value-by-visit interaction as fixed covariates, and assuming an unstructured covariance structure. The model was run separately by treatment group (sarilumab 200 mg q2w and placebo)

*ACR* American College of Rheumatology

*C1M* collagen type I MMP-cleaved fragment, *C2M* collagen type II MMP-cleaved fragment, *CRP* C-reactive protein, *DAS28-CRP* 28-joint disease activity score by CRP, *LDA* low disease activity, *MMP* matrix metalloproteinase, *MTX* methotrexate, *OPG* osteoprotegerin, *q2w* every 2 weeks, *sRANKL* soluble receptor activator of nuclear factor-kB ligand. *Nominal *p* < 0.05 vs nonresponder. **Nominal *p* < 0.01 vs nonresponder

ACR50 responders receiving placebo demonstrated greater reductions in C1M, sRANKL, and the log sRANKL/OPG ratio at week 24 compared with placebo-treated patients who did not achieve ACR50. Other biomarkers suppressed by sarilumab treatment (e.g., C3M) did not significantly differ by ACR50 response.

### Biomarker changes by LDA status at week 24

Serum concentrations of biomarkers were also examined in patients who achieved LDA (placebo, n = 37 (28.9 %); sarilumab 200 mg q2w, n = 72 (55.0 %)) and those who did not achieve LDA (placebo, n = 91 (71.1 %); sarilumab 200 mg q2w, n = 59 (45.0 %)) at week 24 in part B (Table 4). Suppression of CRP according to LDA status was similar to that observed according to ACR50 response. Placebo-treated patients who achieved LDA demonstrated a greater reduction in CRP compared with patients who did not achieve LDA at week 24 (−31.9 % vs −4.2 %; nominal *p* < 0.01) only. As with the ACR50 response analysis, the magnitude of CRP suppression observed in patients who achieved or did not achieve LDA after sarilumab treatment was only slightly different (−96.9 % vs −90.2 %; nominal *p* < 0.01).

**Table 4:** Median percent change from baseline in biomarker concentration in patients who achieved or did not achieve LDA (DAS28-CRP <3.2) at week 24

	Placebo		Sarilumab 200 mg q2w	
	LDA achieved (n = 37)	LDA not achieved (n = 91)	LDA achieved (n = 72)	LDA not achieved (n = 59)
CRP				
Week 2	1.2	−2.9	−95.0^**^	−83.5
Week 24	−31.9^**^	−4.2	−96.9^**^	−90.2
C1M				
Week 2	4.5	2.2	−55.8^*^	−45.1
Week 24	−16.8^*^	−4.3	−65.7^**^	−54.1
C2M				
Week 2	0	3.4	0^*^	−14.3
Week 24	0	3.5	0	−6.7
MMP-3				
Week 2	−2.0	2.3	−7.2	−3.3
Week 24	−9.7	0.4	−47.2	−34.2
OPG				
Week 2	−5.1	1.6	−6.5^*^	−0.9
Week 24	−1.9	−1.8	−4.6	0.9
sRANKL				
Week 2	−4.7	−0.8	−7.9	−2.3
Week 24	−16.2^*^	−0.8	−39.7	−25.8

Percent change from baseline in biomarkers transformed in rank was compared between responder and nonresponder patients at week 24 using an analysis of variance (ANOVA)-type method, with response, visit, and response-by-visit interaction as fixed effects, rank-transformed baseline biomarker value and rank-transformed baseline biomarker-value-by-visit interaction as fixed covariates, and assuming an unstructured covariance structure. The model was run separately by treatment group (sarilumab 200 mg q2w and placebo).

*C1M* collagen type I MMP-cleaved fragment, *C2M* collagen type II MMP-cleaved fragment, *CRP* C-reactive protein, *DAS28-CRP* 28-joint disease activity score by CRP, *LDA* low disease activity, *MMP* matrix metalloproteinase, *MTX* methotrexate, *OPG* osteoprotegerin, *q2w* every 2 weeks, *sRANKL* soluble receptor activator of nuclear factor-kB ligand. *Nominal *p* < 0.05 vs nonresponder. **Nominal *p* < 0.01 vs nonresponder

Trends for reductions in MMP-3, OPG, and sRANKL were observed in both treatment groups between patients who achieved LDA compared with patients who did not. Most of the differences were not significant with the exception of C1M, which was reduced in placebo-treated and sarilumab-treated patients. C3M reduction was not different between patients who did or did not achieve LDA, despite suppression by sarilumab treatment (data not shown).

## Discussion

The effects of treatment with sarilumab plus MTX on biomarkers of joint and tissue destruction and bone resorption were examined in MTX-IR patients with RA from the MOBILITY trial. Given the reduction in the progression of structural damage observed in patients receiving sarilumab 150 or 200 mg q2w (particularly in those receiving 200 mg q2w) [24], blockade of IL-6Rα with this antibody was predicted to significantly impact serum concentrations of biomarkers of joint and tissue destruction and bone resorption. Consistent with this prediction, sarilumab significantly reduced concentrations of markers of joint inflammation (e.g., C3M and MMP-3) and collagen degradation (C2M) compared with placebo. A rapid reduction in several MMP-generated biomarkers was observed as early as 2 weeks after initiation of sarilumab, was sustained for at least 24 weeks, and was dose dependent. Significant correlations between baseline concentrations of C1M, a marker of soft tissue destruction, have previously been observed with CRP concentrations and structural damage in MTX-IR patients with RA, indicating the potential prognostic utility of this marker [27].

Reductions in MMP-3 (stromelysin-1), a marker of synovial inflammation, were also observed at week 2, with continuing reductions observed at week 24, in patients treated with sarilumab compared with those treated with placebo. MMP-3 is highly elevated in the joint tissue and synovial fluid of patients with RA [19, 28], and higher baseline concentrations of this enzyme are associated with disease activity and radiographic progression, particularly in individuals with early RA (i.e., duration of symptoms <12 months) [19].

Serum concentrations of a separate marker of synovial inflammation, CRPM, were also reduced at weeks 2 and 12 in patients treated with sarilumab compared with placebo. Maximum suppression was observed at week 12 in the sarilumab 150 and 200 mg q2w groups, although the mechanism underlying this reduction remains uncertain. Previous reports from the MOBILITY study have shown that sarilumab significantly decreases CRP [24, 26]; as such, the reduction in CRPM observed in the present study could be due to a decrease in proteolysis and/or a decrease in substrate available for MMP-mediated cleavage.

Sarilumab was also associated with a trend toward an increase in OC, a marker of bone formation, in the MTX-IR patient population. Together, the data in the present report are consistent with other studies, in which blockade of IL-6R with tocilizumab was associated with reduced circulating serum concentrations of MMP-3 and MMP-3-cleaved fragments, including C1M, C2M, C3M, and CRPM, and augmentation of OC [18, 27, 29].

Importantly, this placebo-controlled study reported that an inhibition of IL-6 signaling leads to significant sRANKL reduction in patients with MTX-IR RA, which may indicate a potential mechanism through which inhibition of IL-6 signaling prevents further progression of bone resorption and loss in this patient population. Previous work has shown that RANKL concentration is negatively correlated with bone mineral density (BMD) in patients with RA [30, 31] and can be blocked by anti-RANKL monoclonal antibodies that increase BMD, such as denosumab [32]. Furthermore, in patients with refractory RA who received anti-tumor necrosis factor (TNF) therapy, sRANKL serum concentrations of RANKL have been suggested as potential predictive markers of remission [33].

Sarilumab did not significantly affect serum concentrations of OPG, a decoy receptor for RANKL that negatively regulates osteoclast maturation, compared with placebo [34]. This is in contrast with previous observations, in which patients with RA who had received treatment with tocilizumab demonstrated enhanced bone marrow OPG expression relative to patients with RA who had not received biologic therapy [35]. However, in the present analysis of patients with moderate-to-severe RA, blockade of IL-6Rα with sarilumab significantly decreased the sRANKL/OPG ratio, which is often used to measure the magnitude of bone resorption [16] and has been shown to predict 5-year and 11-year joint damage in patients with early untreated RA [16, 36]. The current data also support and expand upon previous work, in which blockade of IL-6R with tocilizumab significantly impacted bone resorption, particularly in patients achieving remission or low disease activity [37].

Although sarilumab significantly suppressed one marker of bone resorption, sRANKL, sarilumab did not significantly modulate CTX-1 relative to placebo at either time point examined. In a previous investigation, treatment with tocilizumab had variable effects on serum concentrations of CTX-1 [18]. In the present study, serum concentrations of CTX-1 were measured at baseline, week 24, and week 52; as there may be a temporal relationship between IL-6Rα blockade and CTX-1 suppression, further analysis at earlier time points may be warranted. CTX-1 is created through cathepsin K cleavage of collagen type I [20, 38]. The lack of CTX-1 modulation observed in the present study suggests that sarilumab may not impact cathepsin K cleavage of collagen type I and may only impact MMP cleavage as reflected in reduction of C1M.

Although posttreatment differences were observed in several biomarkers according to clinical response, most of the significant differences relating to ACR50 response were noted in the placebo group. Modest differences were observed earlier in responders to sarilumab treatment. Additional analysis is needed to determine if the baseline biomarkers or changes in biomarkers can predict clinical response to sarilumab.

The present investigation retrospectively evaluated serum biomarker concentrations collected as part of two independent, randomized, placebo-controlled trials, in which patients with RA received placebo or sarilumab with concomitant MTX. This design not only permitted direct comparison between treatment groups but also allowed for analysis of duration of biomarker responses. This study design also provided a unique opportunity to replicate biomarker assessments in two independent cohorts of similar populations of MTX-IR patients with moderate-to-severe, active RA. The reductions observed in markers of tissue destruction (C1M), cartilage destruction (C2M), and synovial inflammation (C3M) in part A were also observed in part B. Evaluation of joint damage progression in MOBILITY part B provided the basis to also assess biomarkers of bone resorption (CTX-1, OPG, sRANKL, and sRANKL/OPG ratio) and formation (OC). Future analysis will involve correlation of these markers with the radiographic efficacy endpoints (i.e., mTSS, ES, JSN) measured in the main study.

Despite these advantages, there are several limitations. First, only circulating markers of joint damage and resorption were examined. Future studies are needed to examine the effect of sarilumab levels on these markers in the synovial fluid or in synovial tissue. Second, biomarkers were evaluated at a limited number of time points after treatment. Dose-dependent effects observed in MOBILITY part A were not evaluated in MOBILITY part B, which only evaluated the effects of the sarilumab 200 mg q2w dose. Finally, the current analyses do not take into consideration concomitant medications (e.g., corticosteroids [24]) that may possibly influence expression of biomarkers examined in the present study (e.g., RANKL [39]). Therefore, further analyses are needed to clarify the effect on these biomarkers of sarilumab vs concomitant medication vs their combination.

These data are consistent with previous studies and provide additional evidence of reduction in markers of bone resorption in response to sarilumab treatment. Overall, the study findings support a model wherein IL-6 signaling inhibition reduces osteoclast-driven structural damage and reduces joint inflammation markers in patients with established RA and MTX-IR.

## Conclusions

The present investigation demonstrated the pharmacodynamic effects of sarilumab plus MTX on serum concentrations of biomarkers associated with joint and tissue destruction and damage compared with placebo plus MTX. In the future, quantitative measurements of biomarkers of bone and cartilage damage may be useful as prognostic markers to identify patients most in need of treatment. Additionally, such measurements may also serve as predictive markers of positive responses to IL-6 inhibition, allowing interpretation of potential treatment efficacy earlier than radiological-based measurements [40]. To explore the potential role of biomarkers of joint and tissue destruction and inflammation in therapeutic targeting, additional studies are necessary to determine whether serum concentrations of these biomarkers of joint damage, bone resorption, and synovial inflammation can serve as early identifiers of both severe disease and patients likely to respond to sarilumab.

## Abbreviations

ACR: American College of Rheumatology
ANOVA: analysis of variance
C1M: collagen type I matrix metalloproteinase-cleaved fragment
C2M: collagen type II matrix metalloproteinase-cleaved fragment
C3M: collagen type III matrix metalloproteinase-cleaved fragment
CRP: C-reactive protein
CRPM: C-reactive protein matrix metalloproteinase-derived fragment
CTX-1: carboxy-terminal collagen crosslinks 1
DAS28-CRP: 28-joint disease activity score by C-reactive protein
ELISA: enzyme-linked immunosorbent assay
ES: erosion score
FLS: fibroblast-like synoviocytes
IL-6: interleukin 6
IL-6R: interleukin 6 receptor
JSN: joint space narrowing
LDA: low disease activity
MMP: matrix metalloproteinase
mTSS: van der Heijde modified total Sharp score
MTX: methotrexate
MTX-IR: inadequate response to methotrexate
OC: osteocalcin
OPG: osteoprotegerin
q2w: every 2 weeks
RA: rheumatoid arthritis
RANKL: receptor activator of nuclear factor-kB ligand
sRANKL: soluble receptor activator of nuclear factor-kB ligand
TNF: tumor necrosis factor
